# VAR consultation patterns and their association with fouls and misconduct: An analysis of the top five European football leagues

**DOI:** 10.1371/journal.pone.0334518

**Published:** 2025-10-24

**Authors:** Mohamad Nizam Nazarudin, Anwar P. P. Abdul Majeed, Rabiu Muazu Musa, Naresh Bhaskar Raj, Garry Kuan, Noor Azuan Abu Osman

**Affiliations:** 1 Center for the Study of Education and Community Wellbeing, Universiti Kebangsaan Malaysia, Selangor, Malaysia; 2 Faculty of Engineering and Technology, Sunway University, Selangor, Malaysia; 3 Centre for Fundamental and Continuing Education, Universiti Malaysia Terengganu, Terengganu, Malaysia; 4 Faculty of Health Science, School of Rehabilitation Science, Universiti Sultan Zainal Abidin, Gong Badak Campus, Terengganu, Malaysia; 5 Exercise and Sports Science Programme, School of Health Sciences, Universiti Sains Malaysia, Kelantan, Malaysia; 6 Department of Biomedical Engineering, Faculty of Engineering, University of Malaya, Kuala Lumpur, Malaysia; Government Law College, INDIA

## Abstract

Delays and controversies surrounding Video Assistant Referee (VAR) consultations have raised concerns in European football, particularly regarding the types of infractions that prompt referee interventions. This study analysed referee data from 6,232 matches across five seasons in the top five European leagues to identify the foul and misconduct behaviours most strongly associated with VAR referrals. Using clustering and logistic regression, we found that a limited set of offences, most notably handball, off-the-ball challenges, professional fouls, and simulation, were consistently linked to higher consultation frequency. While descriptive comparisons suggested some variation between leagues, league affiliation itself was not a significant predictor once foul type was considered. The findings indicate that VAR is predominantly engaged for offences that are both subjective and potentially decisive in match outcomes. These insights have practical implications for referees, coaches, and players by highlighting the need for strategies that minimise unnecessary consultations, improve game flow, and enhance the consistency of officiating in elite football.

## Introduction

The integration of technology in football has grown substantially since the introduction of goal-line technology in the 2012 FIFA World Cup. Today, tracking systems such as Global Positioning System (GPS) technology are widely used to monitor player movement, quantify performance metrics, and enhance tactical decision-making [1,2]. These systems allow for the measurement of variables such as running speed, total distance covered, field position, heart rate, and workload. In addition to performance tracking, technological advancements have also been extended to officiating, with the International Football Association Board (IFAB) approving the use of the Video Assistant Referee (VAR) system on March 3, 2018, at its 132nd Annual General Meeting [3]. It is worth noting that the primary objective of VAR is to improve refereeing accuracy by assisting on-field officials in making critical match decisions [4].

Despite its intended benefits, the introduction of VAR has sparked significant debate regarding its effectiveness and impact on the game. This motivated researchers to evaluate VAR’s performance in order to determine its strengths and drawbacks. A recent study observed the utilisation of the VAR system in the Israeli Premier League across 212 games. Their analysis identified 89 major mistakes. This number was larger than that of prior years, which was ascribed to the fact that VAR was employed for the first time, and as a result, more crucial match occurrences were recognised [5]. In a different study, an extensive analysis of 9,732 VAR reviews across 13 leagues recorded 795 match-changing incidents, with VAR interventions improving the accuracy of decisions from 92.1% to 98.3% [6]. Research indicates that the introduction of VAR has led to a decline in the frequency of offsides, fouls, and yellow cards, suggesting that players may have adjusted their behaviour to avoid potential VAR scrutiny [7].

While some studies highlight VAR’s ability to enhance fairness and reduce officiating errors [8,9], others emphasise its unintended consequences, such as disruptions in match flow, increased delays, and inconsistencies in referee decision-making [10]. These findings suggest that while VAR enhances decision-making accuracy, its implementation is not without complications, particularly concerning player bookings or dismissals [11]. Consequently, despite the growing body of literature on VAR, few studies have systematically examined the specific types of player infractions that most frequently trigger VAR consultations, particularly across multiple leagues. Most existing research has focused on general decision accuracy, fan sentiment, or the psychological impact on referees [12–14]. This has left a gap in understanding the tactical and misconduct-related actions that prompt VAR involvement. Moreover, cross-league comparative analyses remain scarce, despite evidence suggesting that cultural and contextual factors may influence refereeing styles and interpretations of misconduct [15]. Differences in officiating culture, match tempo, tactical preferences, and disciplinary norms may shape both the frequency and nature of VAR consultations [16]. For instance, leagues such as Serie A and La Liga have historically reported higher foul rates and more stoppages, potentially fostering greater reliance on VAR, while the Premier League has emphasized match flow and minimal intervention. By systematically comparing the top five European leagues, this study captures the extent to which contextual heterogeneity influences VAR usage, providing a more nuanced and generalisable understanding of its operational dynamics. This study addresses these gaps by systematically analysing the types of infractions that lead to VAR consultations across multiple European football leagues. The findings aim to inform referee training, enhance consistency in officiating, and optimise the strategic application of VAR in elite football.

Moreover, to fully understand the impact of VAR interventions on football decision-making, especially in tactical and misconduct-related scenarios, further research is needed. Understanding certain tactical and misconduct-related infractions that require VAR involvement can considerably improve the efficiency of field referee officiating duties. Although the rules are intended to objectively standardise what constitutes a foul, referees often rely on subjective determinations of unusual events in real-time. Similarly, disparities in officiating styles remain evident, as referees demonstrate varying levels of stringency in enforcing the rules, leading to inconsistencies in foul calls and disciplinary sanctions [17]. Furthermore, cross-national studies have revealed differences in caution rates based on referees’ nationalities, implying that cultural influences may shape officiating tendencies and interpretations of misconduct [18]. These variations demonstrate the complexity of refereeing in elite football and highlight the need for a deeper examination of VAR-triggered interventions in tactical and misconduct-related scenarios.

Specifically, in this investigation, we examine the tactical offences and misconduct actions that trigger VAR intervention during European Football League matches. Similarly, the study seeks to determine whether a variation exists across different leagues with respect to the consultations of VAR on the tactical and misconduct actions. We envisage that recognising these actions will enhance the quality of officiating by pinpointing areas where on-field referees should focus more attention or could benefit from additional training. Given the ongoing debate surrounding VAR, these insights are crucial for refining its application and optimising its integration into professional football officiating.

## Methodological approach to the problem

### Data curation

This investigation utilised data from InStat Scout, a subscription-based provider of professional match analytics used by clubs and federations worldwide. InStat created and traced many indicators for every referee, assuring their reliability and validity by tying them to videos of the referee’s activity data and full information on their complete game action (https://instatscout.com/login). To further ensure data validity, the InStat data was compared against data from another football site (https://www.football-data.co.uk/). Specifically, we compared match-level statistics, such as the number of fouls, yellow cards, and red cards recorded in both databases and found a high degree of consistency (>95% match). By doing so, data for 94 matches (1.5%) were removed for incomplete offence count. This process confirmed the accuracy of the data before analysis. The final dataset for this study, therefore, consisted of 6,232 matches from five straight seasons in the English Premier League, Spanish La Liga, Italian Serie A, French Ligue 1, and German Bundesliga.

### Ethical considerations

This study utilised secondary data, obviating the need for explicit participant consent. Secondary data, by definition, are pre-existing and anonymised, thus not directly involving human subjects [19]. Consequently, ethical approval was unnecessary. All data handling and analysis procedures adhered to ethical standards to maintain the integrity and confidentiality of the information used in this research.

### Data characteristics and pre-processing

The dataset included 15 sorts of tactical offences and misconduct activities, consisting of air challenges, ground challenges, dangerous play, misconduct, challenges off the ball, and attack wrecking. A foul is defined as a player’s breach of game rules that inhibits active play and results in a free or penalty kick awarded to the other team. Misconduct denotes incidents requiring disciplinary action, such as dissent, simulation, and unsporting behaviour, and can ensue even while the ball is not in play. Air challenges include aerial duels and challenges where players attempt to win the ball in the air. Ground challenges incorporate slide tackles, standing tackles, and shoulder-to-shoulder challenges, while dangerous play is actions deemed reckless or potentially harmful, such as high-foot challenges. Challenges off the ball involve fouls or altercations a player makes geared towards disrupting opponents’ play and gaining an advantage without possession of the ball. Attack wrecking encompasses tactical fouls used to prevent goal-scoring opportunities, including professional fouls. Before the full analysis, all variables were standardised across the full dataset using z-scores to ensure comparability and preserve cross-league variance.

### Regression diagnostic tests for binary logistic regression

To ensure the validity of the binary logistic regression model, a multicollinearity diagnostic was conducted using the Variance Inflation Factor (VIF) and Tolerance statistics as shown in Table 1. Two predictors (Fouls and Challenges per Foul) were not considered during the model development due to high VIF values (>5) and low Tolerance (<0.25), indicating significant multicollinearity [20]. The remaining predictors (13 variables) had acceptable VIF values (<3) and tolerance values (>0.6), confirming that multicollinearity was not a significant issue [21]. This step ensured the stability and interpretability of the regression coefficients in the final model. Reducing the predictors from 15 to 13 also helped mitigate redundancy, improve model parsimony, and reduce the risk of inflated standard errors, thereby strengthening the robustness of the findings. Importantly, this refinement allowed the model to focus on the most theoretically and statistically meaningful variables, enhancing both accuracy and clarity of interpretation.

**Table 1:** Multicollinearity diagnostic for the binary logistic regression model.

Predictors	VIF	Tolerance
Fouls	5.33	0.226
Challenges per foul	5.31	0.221
Yellow cards	2.12	0.652
Direct red cards	1.31	0.748
RC for two YC	1.16	0.865
Fouls per card	1.55	0.801
Air challenges	1.19	0.821
Ground challenges	1.71	0.721
Handball	1.34	0.724
Challenge off the ball	1.41	0.718
Dangerous play	1.26	0.786
Misconduct	1.58	0.619
Simulation/Diving	1.21	0.834
Attack wrecking	1.58	0.612
Professional foul	1.23	0.794
League	1.26	0.752

### Data analysis

In the initial stage of data analysis, a cluster analysis was conducted to group referees’ VAR consultations into meaningful clusters, categorised as high or low based on the frequency of consultations across the studied leagues. These clusters were then used to develop a binary multivariate logistic regression model, with VAR consultation levels (high and low) as the dependent variables. The clustering step in this study serves as a conceptual categorisation tool, designed to differentiate between matches characterised by frequent VAR consultations (“high-interference”) and those with minimal involvement (“low-interference”). This binary construct is not an arbitrary threshold but an empirically derived grouping based on the distribution of VAR usage frequencies across all matches in the dataset. The rationale is to operationalise VAR consultation patterns in a way that reflects real-world officiating contexts, enabling the subsequent logistic regression analysis to assess which types of fouls and misconduct statistically predict a match falling into the high-versus low-consultation category. This combined approach allows exploratory (unsupervised) methods to inform predictive (supervised) modelling, while maintaining conceptual coherence between the classification and explanatory objectives. The 13 types of fouls and misconduct offences extracted after conducting the multicollinearity tests served as independent variables, while the leagues were included as covariates to determine any significant variations in referees’ intentions to consult VAR across different leagues. The binary multivariate logistic regression model was deemed appropriate for this study, given the categorical nature of the dependent variable and the different types of foul and misconduct offences as independent variables with the leagues as covariates [22,23]. This model allowed for the estimation of the likelihood of VAR consultation by referees across various leagues. Additionally, a correlation analysis was performed to examine the relationship between fouls and misconduct offences.

Furthermore, a Chi-square analysis was used to study the frequency of referees consulting VAR, regardless of the foul and misconduct offence variables. The Chi-square analysis was chosen because the variables of interest were categorical, i.e., two groups of VAR consultations versus different leagues, as suggested by previous researchers [24,25]. The association between these variables is presented in a radar plot for easier interpretation of the likelihood of on-field referees consulting VAR based on frequencies across different leagues. A detailed description of the statistical analyses applied in the study is provided in the following sub-section.

### Clustering the VAR consultations

K-means clustering is an unsupervised learning approach for partitioning datasets into k clusters. The algorithm selects k centroids (or cluster centres) and allocates each data point to the nearest centroid [26–28]. The centroid is then updated to the average position of the points given to it, and the process continues until the centroids stabilise and no longer fluctuate appreciably. The basic goal of K-means clustering is to reduce the overall distance between data points and their centroids.

In this investigation, we employed a K-means clustering approach to group VAR interventions based on their frequency. This method was chosen for its ability to segment data without predefined categories, allowing for unbiased pattern identification. It is worth noting that the k-means clustering was based solely on the number of VAR reviews per match. The predictors, i.e., foul and misconduct variables, were not used in the clustering process but were entered as predictors in the subsequent logistic regression model as previously applied in referee decision-making research [29]. We utilised the Euclidean distance measure and the silhouette analysis technique to identify group formations. Similarly, an independent t-test was applied to determine the differences between the two cluster and the extracted 13 predictors. This approach provides a clear categorisation of VAR interventions, enabling a deeper exploration of the factors influencing on-field officials’ tendency to consult VAR.

### Development and evaluation of the logistic regression model

A multivariable binary logistic regression model was used to analyse the likelihood of on-field referees consulting VAR based on the extracted 13 types of fouls and misconduct offences. These 13 variables were treated as independent variables, while the two clusters obtained through k-means clustering served as the dependent variables. Moreover, the leagues were used as a covariate to assess whether the relationship between fouls/misconduct as well as VAR intervention, differs by league. In other words, league differences were included as a covariate to test whether referee behaviour varied across competitions. It is important to highlight that the predictor variables in the regression model were selected based on both theoretical and empirical considerations. Theoretically, the *Laws of the Game* identify certain offences such as fouls involving physical contact, handling the ball, simulation, and professional fouls as having the greatest influence on match outcomes and being most likely to necessitate VAR adjudication [30]. Empirically, prior research has consistently identified these categories as among the most frequently reviewed in VAR operations across various leagues [1,9,31]. Including these variables ensures the model is grounded in established officiating principles while also reflecting evidence from recent empirical analyses.

To minimise model complexity and multicollinearity, predictor selection for the binary logistic regression model followed a Forward Stepwise (Likelihood Ratio) approach, which has been used in exploratory sports analytics research to identify the most parsimonious model from a large set of candidate variables [32]. To mitigate risks of overfitting and coefficient instability often associated with stepwise procedures [33], we implemented additional robustness measures in such a way that the regression model was validated using 10-fold cross-validation, ensuring coefficient stability across different data splits. Moreover, the Hosmer–Lemeshow decile plot was applied to compare observed and expected frequencies of high VAR consultations across deciles of predicted probabilities in order to check for systematic over- or under-fitting. This method systematically removes non-significant predictors while maximising model performance. Odds ratio (OR) estimations and their 95% confidence intervals (CI) were used to present the results. We employed Nagelkerke’s R^2^ as an effect size metric to evaluate the quality of the model; values were interpreted as follows: 0.02–0.13 for a little effect, 0.13–0.26 for a medium effect, and more than 0.26 for a high effect [34,35]. The Hosmer-Lemeshow test was applied to evaluate model fit, and the area under the curve (AUC) from Receiver Operating Characteristic (ROC) analysis was used to measure discriminant capacity. Predicted probabilities were used to create the ROC curve for every variable.

It is important to note that in this study, Nagelkerke’s R² was reported as a measure of model fit, following its established use as a rescaled pseudo-R² statistic in logistic regression contexts [36,37]. While it does not represent the proportion of variance explained in the same way as R² in linear regression, it offers a comparative indication of improvement in model fit relative to a null model. Its inclusion in this study is intended to complement other diagnostics such as the Hosmer–Lemeshow test, area under the ROC curve (AUC), and classification accuracy, which together provide a more comprehensive assessment of model performance. Jamovi version 2.4 and Python 3.0 were utilised for all statistical analyses, with a significance level drawn at p ≤ 0.05. This comprehensive approach ensures that the findings are robust and reliable, providing valuable insights into the factors influencing VAR consultations.

## Results

**Fig 1:**
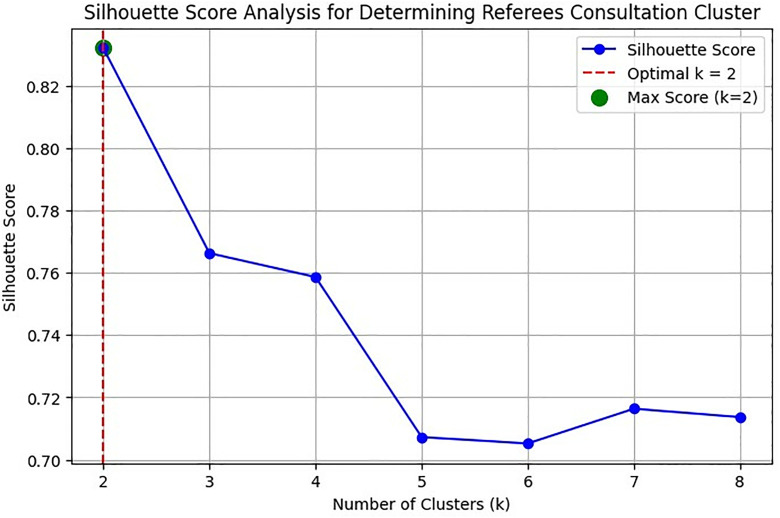
Silhouette plot analysis for determination of referees’ consultation cluster.

**Fig 2:**
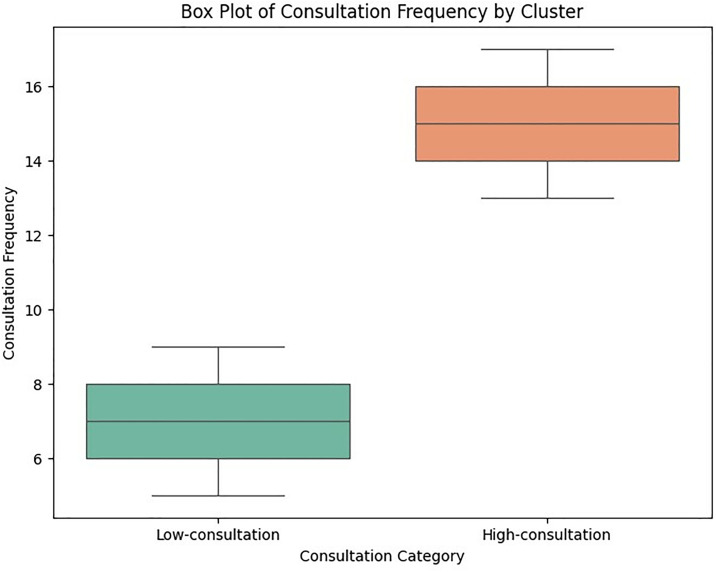
Box-plot of differences between the two referees’ consultation cluster.

Fig 1 illustrates the silhouette scores for different numbers of clusters (k) in a cluster analysis of the consultation dataset. The silhouette score evaluates the clustering quality, with higher values indicating better-defined clusters. The optimal number of clusters is determined at k = 2, where the silhouette score reaches its maximum. The red dashed line marks this optimal k-value, and the green dot highlights the highest silhouette score, demonstrating that two distinct groups provide the best clustering structure for the data. This implies that referees’ consultation can be effectively categorised into two. Meanwhile, the box plot of differences in the frequency of consultation by the referees is depicted in Fig 2. A clear separation could be observed from the Figure, where the high-consultation cluster depicts a considerably higher frequency of VAR engagement for assistance, while the low-consultation cluster demonstrates fewer engagements. Moreover, Table 2 presents the descriptive statistics for each cluster, highlighting significant differences in the mean frequency of the examined offences. These operational labels align with elite match officiating practice, where an accumulation of high-impact incidents tends to escalate VAR engagement, while low-intensity matches generate fewer reviewable situations. In essence, the high-consultation cluster demonstrated matches with frequent and multiple offence-driven VAR use, typically involving incidents such as ground challenges, simulation/diving, attack wrecking, and handball, which required prolonged reviews. Conversely, the low-consultation cluster included matches with minimal or rare VAR involvement, often limited to isolated or low-severity offences that did not demand extended intervention.

**Table 2:** Groups comparisons of fouls and misconduct offences of the VAR interventions.

Predictors	VAR Group		p-value
	High consultation (*n* = 200)	Low consultation (*n* = 402)	
Yellow cards	71.680 ± 19.432	34.756 ± 26.319	0.001*
Direct red cards	1.820 ± 1.842	0.898 ± 1.290	0.001*
RC for two YC	1.720 ± 1.379	0.766 ± 1.085	0.001*
Fouls per card	116.951 ± 38.795	71.993 ± 55.180	0.001*
Air challenges	2.960 ± 2.684	0.939 ± 0.713	0.001*
Ground challenges	18.385 ± 7.119	4.035 ± 4.344	0.001*
Handball	1.195 ± 1.218	0.271 ± 0.573	0.001*
Challenge off the ball	1.385 ± 1.279	0.271 ± 0.559	0.001*
Dangerous play	12.930 ± 7.093	2.420 ± 2.805	0.001*
Misconduct	14.210 ± 6.995	2.978 ± 3.326	0.001*
Simulation/Diving	0.535 ± 0.807	0.142 ± 0.390	0.001*
Attack wrecking	21.545 ± 7.018	4.953 ± 4.766	0.001*
Professional foul	0.835 ± 0.955	0.149 ± 0.409	0.001*

Values are presented as median±standard deviation.

*Significant difference across the two groups (p < 0.01).

Table 3 presents the performance fit of the developed multivariate binary logistic regression model. The results indicate a significant regression, accentuating the importance of the variables in explaining the outcome measures (p < 0.001). Additionally, the model achieved a well-fitting value (Hosmer-Lemeshow > 0.05) and demonstrated strong predictive power with a Nagelkerke R² of 0.95, accounting for approximately 95% of the odds for the occurrences of overall events. This suggests a high likelihood of on-field referees consulting VAR based on the occurrences of fouls and misconduct offences examined. Furthermore, the model shows an AIC of 112 and BIC values of 191, further demonstrating the robustness and reliability of the model.

**Table 3:** Model performance measures.

Deviance	AIC	BIC	Hosmer Lemeshow (p)	Overall Model Test			
				R²N	χ²	df	p
75.6	112	191	0.903	0.948	690	17	0.001

**Fig 3:**
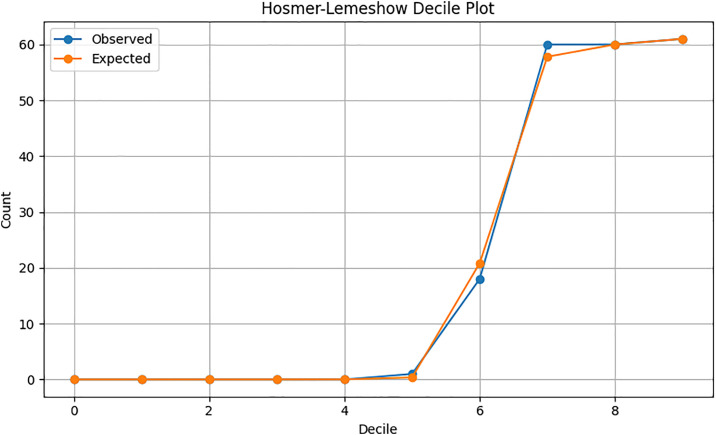
Hosmer-Lemeshow decile plot of observed and expected frequencies of high VAR consultations.

The Hosmer–Lemeshow decile plot comparing observed and expected frequencies of high VAR consultations across deciles of predicted probabilities is demonstrated in Fig 3. The close alignment between observed and expected values across all deciles, particularly in the higher-risk groups (deciles 6–9), suggests that the model is well-calibrated. There is no evidence of systematic over- or under-estimation, indicating a good fit of the logistic regression model to the data.

Table 4 presents the results of the multivariable binary logistic regression model, which aimed to identify significant misconduct and foul-related offences predicting the likelihood of on-field referees consulting VAR. From the analysis, seven out of the 13 offences were found to be statistically significant (p < 0.05). These include ground challenges, handball, challenges off the ball, misconduct, simulation/diving, attack wrecking, and professional fouls. These findings shed light on the factors influencing VAR consultations during matches.

**Table 4:** Significant fouls and misconduct offences determining the degree of on-field referees consulting VAR.

Predictors	*B*	SE	Z	p	Odds ratio	95% Confidence Interval	
						Lower	Upper
Intercept	17.118	3.266	5.242	0 .001	2.700	4.504	1.641
Yellow cards	0.026	0.050	0.512	0.609	1.026	0.930	1.133
Direct red cards	0.105	0.332	0.317	0.752	1.111	0.580	2.128
RC for two YC	−0.045	0.366	−0.124	0.901	0.956	0.467	1.957
Fouls per card	−0.016	0.010	−1.537	0.124	0.984	0.964	1.004
Air challenges	−0.573	0.365	−1.569	0.117	0.564	0.275	1.154
Ground challenges	−0.296	0.092	−3.207	0.001*	0.744	0.620	0.891
Handball	−1.453	0.499	−2.913	0.004*	0.234	0.088	0.622
Challenge off the ball	−1.475	0.454	−3.252	0.001*	0.229	0.094	0.557
Dangerous play	−0.155	0.102	−1.511	0.131	0.857	0.701	1.047
Misconduct	−0.344	0.123	−2.791	0.005*	0.709	0.557	0.903
Simulation/Diving	−1.584	0.517	−3.062	0.002*	0.205	0.074	0.565
Attack wrecking	−0.289	0.088	−3.273	0.001*	0.749	0.630	0.891
Professional foul	−1.225	0.500	−2.451	0.014*	0.294	0.110	0.783
**League:**							
Bundesliga – Premier	−1.170	1.362	−0.859	0.391	0.31	0.0215	4.476
Laliga – Premier	−0.576	2.074	−0.278	0.781	0.562	0.0097	32.777
League1 – Premier	−0.438	1.684	−0.26	0.795	0.645	0.0238	17.517
SeriesA – Premier	−1.132	1.606	−0.705	0.481	0.322	0.0139	7.505

Note: *p < 0.05; SE = Standard Error; OR = Odd Ratio

Specifically, for every decrease in the occurrence of ground challenges, the chances of on-field referees consulting VAR reduce by 26% (OR = 0.744, CI95% = [0.620–0.891]). A decrease in handball incidents leads to a 77% reduction in VAR consultations (OR = 0.234, CI95% = [0.088–0.622]). Similarly, a decrease in committing challenges off the ball, misconduct, simulation/diving, attack wrecking, and professional fouls decreases the odds of on-field referees consulting VAR by 77%, 29%, 78%, 25%, and 71% respectively (OR = 0.229, CI95% = [0.094–0.557]; OR = 0.709, CI95% = [0.557–0.903]; OR = 0.215, CI95% = [0.076–0.603]; OR = 0.749, CI95% = [0.630–0.891]; OR = 0.294, CI95% = [0.110–0.783]).On the other hand, the analysis of league-specific effects revealed that none of the league coefficients were statistically significant (p > 0.05), indicating no clear evidence that any particular league influences the likelihood of VAR consultation when controlling for the included misconduct and foul-related offenses.

**Fig 4:**
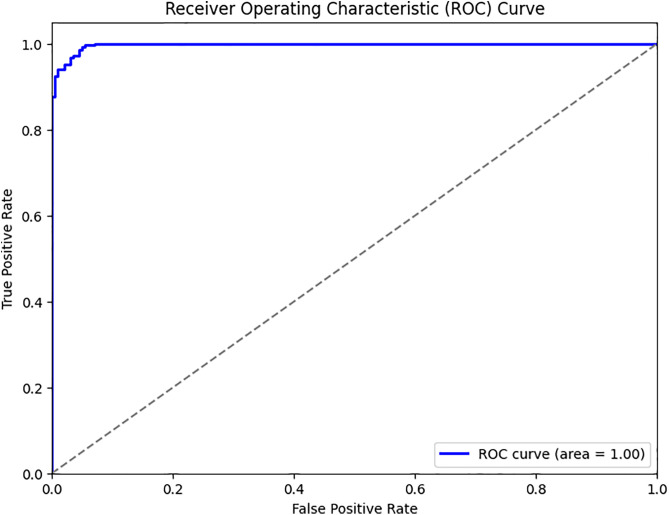
Model performance based on ROC curve analysis.

Fig 4 depicts the model performance based on ROC curve analysis. The predictive model achieved a mean accuracy score of 97%, indicating a strong ability to predict referees’ consultations. The Area Under the Curve (AUC) was 0.99, signifying excellent modelling in predicting the likelihood of on-field referees consulting VAR. The figure further indicates that the sensitivity and specificity scores were 0.96 and 0.98, respectively. These scores revealed that the model predicted more than 96% of positive cases and correctly identified 98% of the actual positive classes, demonstrating that the model predicted both true positives and true negatives with high precision, effectively distinguishing between instances where VAR consultation was necessary and where it was not.

Table 5 shows the classification matrix of the developed model after cross-validation. This technique was employed to evaluate the performance of the logistic regression (LR) model in predicting the likelihood of on-field referees consulting VAR. The model correctly predicted 192 out of 200 high-consultations, indicating only 8 misclassifications. Additionally, the model accurately predicted 393 low-consultations out of 402, with only 9 misclassifications. Overall, it is apparent that the model performed well in the classification task, demonstrating strong accuracy and reliability in distinguishing between high and low consultations based on the data studied.

**Table 5:** Classification table for the model developed.

Observed	Predicted		
	High-consultation	Low-consultation	% Correct
High-consultation	192	8	96
Low-consultation	9	393	97.8

**Fig 5:**
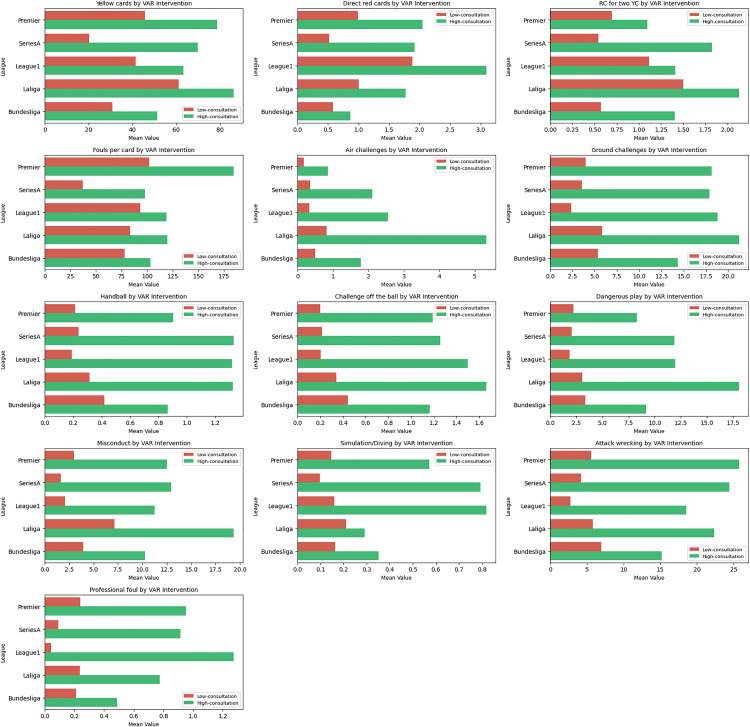
Variation in VAR consultation within each league based on various foul and misconduct offences.

Fig 5 presents the differences in VAR consultation within leagues based on various foul and misconduct offences. The bar plots compare the mean values of these infractions between high and low VAR consultation cases in the leagues involved, i.e., Premier League, Serie A, Ligue 1, La Liga, and Bundesliga. It could be observed from the Figure that high VAR consultation cases are associated with higher mean values for most misconduct-related variables, particularly for ground challenges, challenges off the ball, misconduct, simulation/diving, attack wrecking, and professional fouls within each league.

**Fig 6:**
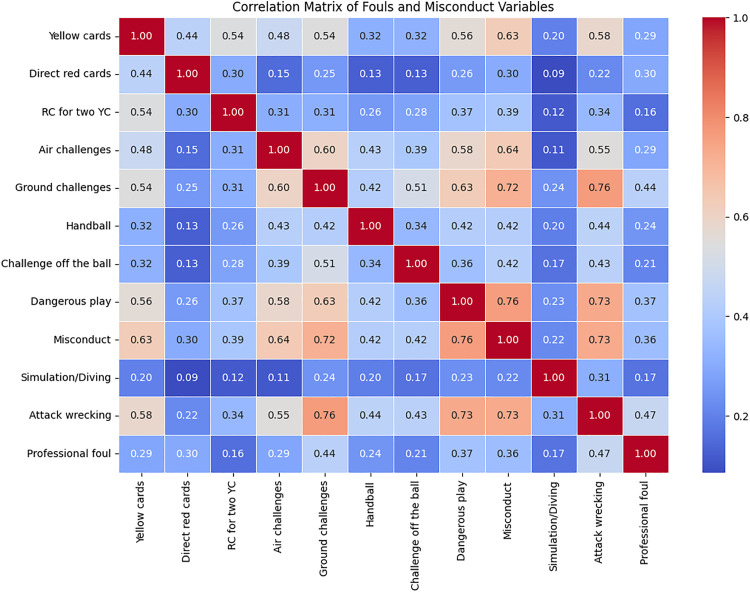
Correlation matrix among various fouls and misconduct-related offences.

Fig 6 depicts a correlation matrix of relationships among various fouls and misconduct-related variables in football, revealing key patterns in player behaviour and disciplinary actions. Strong correlations were observed between ground challenges and misconduct (r = 0.72), as well as dangerous play and misconduct (r = 0.76), indicating that frequent fouling and aggressive challenges often lead to disciplinary measures. Additionally, attack wrecking and ground challenges (r = 0.76) suggest that defensive aggression contributes to misconduct. Moderate correlations were found between yellow cards and misconduct (r = 0.63), and professional foul and attack wrecking (r = 0.47), emphasising the link between disruptive play and disciplinary actions. Conversely, simulation/diving showed weak correlations with other variables, suggesting it operates independently of other misconduct behaviours. Similarly, direct red cards appeared to result from isolated incidents rather than patterns of aggressive or disruptive play. Overall, these findings suggest that foul frequency, aggressive challenges, and misconduct are closely interrelated, whereas simulation and direct red cards exhibit distinct behavioural patterns.

**Fig 7:**
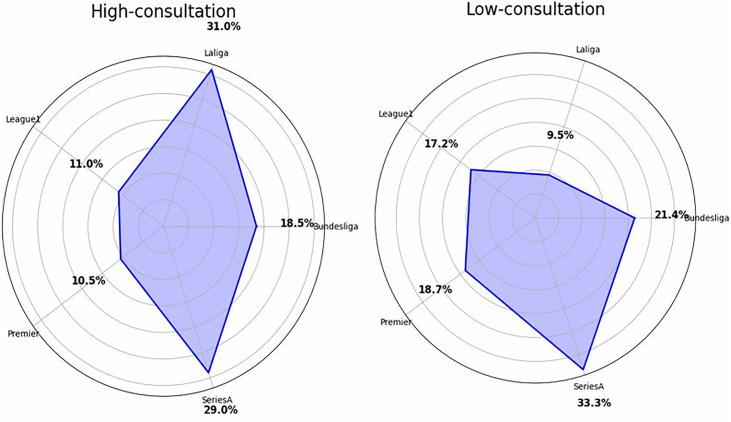
Comparative analysis of VAR consultation frequency across different leagues.

Fig 7 demonstrates the radar charts generated via the Chi-square analysis that illustrate the distribution of VAR consultations across different football leagues, comparing high-consultation and low-consultation scenarios irrespective of fouls or misconduct offences. In the high-consultation scenario, La Liga (31.0%) and Serie A (29.0%) show the highest reliance on VAR, followed by Bundesliga (18.5%), Ligue 1 (11.0%), and the Premier League (10.5%). Conversely, in the low-consultation scenario, Serie A (33.3%) and Bundesliga (21.4%) exhibit the highest probability of minimal VAR involvement, followed by the Premier League (18.7%), Ligue 1 (17.2%), and La Liga (9.5%). Overall, the results suggest that La Liga and Serie A tend to engage in frequent VAR reviews, whereas the Premier League and Ligue 1 show a relatively balanced distribution between high and low consultations. Bundesliga demonstrates moderate reliance on VAR in both scenarios. It is worth noting that these frequencies in VAR consultation across the leagues are not reliant upon misconduct and foul-related offences but rather focus solely on the number of times VAR are consulted regardless of any specific incidents.

## Discussion

The main purpose of this study was to identify specific fouls and misconduct offences that significantly predict Video Assistant Referee (VAR) consultations in elite European football. By examining over 6,000 matches across the top five leagues, the study aimed to clarify which infractions most frequently trigger VAR interventions and assess whether these patterns vary across leagues.

The multivariate binary logistic regression model indicated a significant regression, highlighting the importance of the variables in explaining the outcome measures (Table 3 and Fig 4). This finding aligns with recent literature that emphasises the value of statistical modelling for understanding complex decision-making in officiating [38,39]. The model demonstrated excellent fit, with a Nagelkerke R^2^ of 0.95, suggesting strong explanatory power beyond the null model [40,41]. Comparable applications in sports analytics have shown that high pseudo-R^2^ values, alongside robust classification and validation metrics, provide credible evidence of model performance [34,42].

The results indicate that tactical and misconduct-related offences such as ground challenges, challenges off the ball, misconduct, simulation/diving, attack wrecking, and professional fouls are most strongly associated with VAR interventions [43]. Misconduct can occur at any stage of the game and remains difficult to monitor [44,45]. As football has grown faster and more aggressively, these behaviours have become more prevalent [46–48], increasing referees’ cognitive demands and error risks [49]. Stricter enforcement of dangerous tackles by FIFA has further necessitated VAR use [3]. Consequently, it is not surprising that offences like misconduct, attack wrecking, and ground challenges frequently attract VAR review, accentuating the need for referee training in these contexts.

Aggressive play and fouls have contributed to excessive delays in recent seasons, with ground challenges and handballs among the most contentious and frequently reviewed incidents [11]. Minimising such offences can reduce interruptions and psychological strain on referees and players [50]. Consequently, this effort could reduce pressure from referees as a result of heightened scrutiny during VAR reviews [51]. Targeted training and rule adjustments may therefore improve officiating efficiency and spectator experience.

These findings can also be interpreted through decision-making theory under uncertainty [52], which suggests referees depend on both perceptual cues and contextual knowledge when adjudicating ambiguous situations. VAR acts as an external decision aid that reduces cognitive load but alters decision flow [53]. Infractions such as handball and simulation that are prone to ambiguity and contestation are especially likely to trigger technological assistance, highlighting the trade-off between fairness and match flow.

As shown in Fig 5, VAR consultation rates varied across leagues in exploratory analyses, with higher consultation cases linked to misconduct-related offences such as ground challenges, simulation, and professional fouls [1]. Correlations presented in Fig 6 further showed strong links between ground challenges, misconduct, dangerous play and misconduct, highlighting that referees consistently penalise repeated infractions [9]. This has further reinforced the association between aggressive play and disciplinary action as reported in a previous investigation [31]. Moderate correlations (e.g., attack wrecking with ground challenges, or professional foul with attack wrecking) suggest defensive aggression as a key factor in disrupting play [54], while weak correlations observed in simulation/diving with direct red cards highlight subjectivity in these decisions [55].

Although the regression model revealed no statistically significant league-specific effects (p > 0.05), Fig 7 illustrates trends suggesting differences in officiating philosophies and VAR reliance. For example, leagues like the Premier League emphasise continuity with a higher threshold for intervention, whereas La Liga and Serie A appear more interventionist [56]. These patterns may reflect cultural and institutional factors [54,55], with the “clear and obvious error” principle contrasting with lower tolerance for body contact in other leagues [18]. Nonetheless, the absence of direct procedural metrics limits firm causal claims. Future research could investigate referee training and institutional directives to explain these contextual variations explicitly.

Overall, the findings indicate that VAR is disproportionately engaged for subjective but consequential offences. For players and coaches, this highlights the importance of adapting tactics to minimise handball, simulation, and off-the-ball challenges. Coaches may integrate controlled tackling drills and video-based feedback to reduce contentious incidents. For referees, targeted training can improve recognition of misconduct and tactical fouls, ensuring consistency. At the governance level, refining VAR protocols such as standardising thresholds for offence categories could enhance transparency, efficiency, and confidence in officiating.

### Study limitation

This study has several limitations that should be acknowledged. First, the analysis is observational, which limits the ability to establish causal relationships between offense types and VAR consultations. Potential confounding factors such as match importance, rivalry intensity, home–away status, and referee experience were not included in the models but could influence decision-making patterns. Second, the dataset was restricted to the five top European leagues, which may limit generalisability to other competitions with different officiating cultures or VAR protocols. Although our results showed no statistically significant league-specific effects on VAR consultations when controlling for misconduct and foul-related offences, future research could explore whether subtle contextual differences exist across leagues beyond those captured in our model. Additionally, this study does not account for the potential feedback loop in which the presence of VAR itself may influence player behaviour, either by increasing caution in certain situations or encouraging more strategic exploitation of the system, such as simulation. As VAR continues to evolve, future research should consider match-specific variables such as referee experience, crowd noise intensity, and game importance (e.g., relegation battles vs. mid-season matches) as potential confounders influencing VAR reliance. Moreover, further studies should examine its long-term impact on player conduct, referee decision-making, and overall game dynamics.

## Conclusion

The findings of this study demonstrated that seven types of fouls and misconduct offences, comprising ground challenges, handball, challenges off the ball, misconduct, simulation/diving, attack wrecking, and professional fouls, are significant predictors of high VAR consultation matches. Reductions in these incidents were associated with decreases in VAR usage ranging from 24% to 78%. These patterns, consistent across the top five European leagues, suggest that VAR is disproportionately triggered by specific forms of unsporting behaviour rather than routine tactical infringements. Notably, these offences also demonstrate strong associations with VAR interventions, accentuating the role of disciplinary and tactical infractions in triggering video review.

### Recommendation and future directions

The overall insights from the current investigation accentuate the need for referee training programs to incorporate targeted strategies for identifying and managing these high-impact situations effectively. By enhancing referees’ ability to anticipate and address offences that frequently lead to VAR reviews, such training can improve decision-making efficiency, minimise match disruptions, and ensure a more fluid and fair game. Additionally, integrating VAR-related scenarios into referee development programs can better equip officials, particularly new referees, to handle in-game challenges, ultimately fostering consistency and confidence in officiating across different leagues. Future research should also investigate the cultural and institutional dimensions of VAR implementation, as well as explore league-specific variations in refereeing philosophies, enforcement thresholds, and protocols for intervention. Such analyses could reveal how differing interpretations of obvious error, tolerance for physical contact, or pressure from stakeholders influence the frequency and context of VAR usage across leagues.

## Supporting information

S1 File Raw Dataset used in the study. (XLSX)

## References

[1] de Oliveira MS, Steffen V, Trojan F. A systematic review of the literature on video assistant referees in soccer: challenges and opportunities in sports analytics. Decis Anal J. 2023;7:100232.

[2] Carling C, Bloomfield J, Nelsen L, Reilly T. The role of motion analysis in elite soccer. Sport Med. 2008;38:839–62. 10.2165/00007256-200838100-00004 18803436

[3] International Football Association Board (IFAB). Statutes of The International Football Association Board [Internet]. Zurich: IFAB; 2021 [cited 2025 Sep 17]. Available from: https://www.theifab.com/

[4] Teixeira da Silva JA, Nazarovets S, Carboch J, Deutscher C, Almeida CH, Webb T. The video assistant referee in football. Sport Eng. 2024;27:14.

[5] Samuel RD, Galily Y, Filho E, Tenenbaum G. Implementation of the Video Assistant Referee (VAR) as a career change-event: The Israeli Premier League Case Study. Front Psychol. 2020;11:564855. doi: 10.3389/fpsyg.2020.564855 33224057 PMC7668198

[6] Spitz J, Wagemans J, Memmert D, Williams AM, Helsen WF. Video assistant referees (VAR): the impact of technology on decision making in association football referees. J Sports Sci. 2021;39(2):147–53. doi: 10.1080/02640414.2020.1809163 32794432

[7] Carlos L-P, Ezequiel R, Anton K. How does Video Assistant Referee (VAR) modify the game in elite soccer? Int J Perform Anal Sport. 2019;19(4):646–53. doi: 10.1080/24748668.2019.1646521

[8] Dufner A-L, Schütz L-M, Hill Y. The introduction of the Video Assistant Referee supports the fairness of the game - an analysis of the home advantage in the German Bundesliga. Psychol Sport Exerc. 2023;66:102386. doi: 10.1016/j.psychsport.2023.102386 37665851

[9] Gasparetto T, Loktionov K. Does the Video Assistant Referee (VAR) mitigate referee bias on professional football? PLoS One. 2023;18(11):e0294507. doi: 10.1371/journal.pone.0294507 38011199 PMC10681297

[10] Scanlon C, Griggs G, McGillick C. ‘It’s not football anymore’: perceptions of the video assistant referee by English Premier League football fans. Soccer Soc. 2022;23(8):1084–96. doi: 10.1080/14660970.2022.2033731

[11] Harwood H. Investigating video assistant referees (VARs): Does contextual information influence VAR decision-accuracy and decision-severity in football. Colchester (UK): University of Essex; 2024.

[12] Albanese A, Baert S, Verstraeten O. Twelve eyes see more than eight. Referee bias and the introduction of additional assistant referees in soccer. PLoS One. 2020;15(2):e0227758. doi: 10.1371/journal.pone.0227758 32101548 PMC7043735

[13] Samuel RD, Galily Y, Englert C, Basevitch I. Football referees issue more yellow cards following VAR interventions – mental, tactical, and performance considerations. Int J Sports Sci Coach. 2024;20(1):184–92. doi: 10.1177/17479541241289078

[14] South Z. The usage of video assistant referee (VAR) and its impact on players’ and fans’ emotional experience of football. London (UK): University College London. 2022. pp. 1–92.

[15] Webb T, Rayner M, Cleland J, O’Gorman J. Referees, match officials and abuse: research and implications for policy. 1st ed. London: Routledge; 2020.

[16] Lago-Peñas C, Gómez-López M. The influence of referee bias on extra time in elite soccer matches. Percept Mot Skills. 2016;122(2):666–77. doi: 10.1177/0031512516633342 27166341

[17] Fuller CW, Junge A, Dvorak J. An assessment of football referees’ decisions in incidents leading to player injuries. Am J Sports Med. 2004;32(1 Suppl):17S–22S. doi: 10.1177/0363546503261249 14754855

[18] Dawson P, Dobson S. The influence of social pressure and nationality on individual decisions: Evidence from the behaviour of referees. J Econ Psychol. 2010;31:181–91.

[19] Irwin S. Qualitative secondary data analysis: ethics, epistemology and context. Prog Dev Stud. 2013;13(4):295–306. doi: 10.1177/1464993413490479

[20] Cropper JP. Multicollinearity within selected western North American temperature and precipitation data sets. Edmonton (Canada): University of Alberta; 1984.

[21] Kyriazos T, Poga M. Dealing with multicollinearity in factor analysis: the problem, detections, and solutions. Open J Stat. 2023;13(03):404–24. doi: 10.4236/ojs.2023.133020

[22] Musa RM, Majeed APPA. Sleep duration and its association with physical attributes in young Malaysian adults. Int J Sport Health Sci. 2024;22(0):130–9. doi: 10.5432/ijshs.202322

[23] Glonek GFV, McCullagh P. Multivariate logistic models. J R Stat Soc Ser B. 1995;57:533–46.

[24] Husin Musawi Maliki AB, Mohd Isa AM, Nazarudin MN, Abdullah MR, Mat-Rasid SM, Musa RM. Patterns in assignment submission times: analysis of factors contributing to undergraduate students’ commitment to core-curriculum related course. Heliyon. 2024;10(4):e26214. doi: 10.1016/j.heliyon.2024.e26214 38420391 PMC10900937

[25] Musa RM, Hassan I, Abdullah MR, Latiff Azmi MN, Abdul Majeed APP, Abu Osman NA. A longitudinal analysis of injury characteristics among elite and amateur tennis players at different tournaments from electronic newspaper reports. Front Public Health. 2022;10:835119. doi: 10.3389/fpubh.2022.835119 36033746 PMC9399393

[26] Eswaramoorthi V, Abdullah MR, Musa RM, Maliki ABHM, Kosni NA, Raj NB, et al. A multivariate analysis of cardiopulmonary parameters in archery performance. Hum Mov. 2018;19(4):35–41. doi: 10.5114/hm.2018.77322

[27] Razali MR, Alias N, Maliki A, Musa RM, Kosni LA, Juahir H. Unsupervised pattern recognition of physical fitness related performance parameters among Terengganu youth female field hockey players. Int J Adv Sci Eng Inf Technol. 2017;7:100–5.

[28] Taha Z, Haque M, Musa RM, Abdullah MR, Maliki A, Alias N. Intelligent prediction of suitable physical characteristics toward archery performance using multivariate techniques. J Glob Pharma Technol. 2009;9:44–52.

[29] Musa RM, Majeed AP, Abdullah MR, Kuan GRM. Data mining and machine learning in high-performance sport. 1st ed. Singapore: Springer; 2022.

[30] International Football Association Board (IFAB). Changes to the Laws of the Game 2023/24 [Internet]. Zurich: IFAB; 2023 [cited 2025 Sep 17]. Available from: https://downloads.theifab.com/downloads/lotg_changes_23_24_en

[31] Li M, Wang X, Zhang S. The effect of video assistant referee (VAR) on match performance in elite football: A systematic review with meta-analysis. Proc Inst Mech Eng Part P J Sport Eng Technol. 2024. doi: 17543371241254596

[32] Derksen S, Keselman HJ. Backward, forward and stepwise automated subset selection algorithms: frequency of obtaining authentic and noise variables. Br J Math Stat Psychol. 1992;45:265–82.

[33] Steyerberg EW, Eijkemans MJ, Habbema JD. Stepwise selection in small data sets: a simulation study of bias in logistic regression analysis. J Clin Epidemiol. 1999;52(10):935–42. doi: 10.1016/s0895-4356(99)00103-1 10513756

[34] Carvalho V, Esteves PT, Nunes C, Helsen WF, Travassos B. The assessment of the match performance of association football referees: Identification of key variables. PLoS One. 2023;18(9):e0291917. doi: 10.1371/journal.pone.0291917 37733773 PMC10513314

[35] Sousa H, Clemente FM, Sarmento H, Gouveia ÉR, Musa RM. Comparing locomotor intensity indicators in soccer training and competition across contextual factors: a study of replaced coaches in a Portuguese professional 1st league team. Front Sports Act Living. 2024;6:1391784. doi: 10.3389/fspor.2024.1391784 38854423 PMC11157433

[36] Menard S. Applied logistic regression analysis. 2nd ed. Thousand Oaks (CA): Sage Publications; 2001.

[37] Nagelkerke NJD. A note on a general definition of the coefficient of determination. Biometrika. 1991;78(3):691–2. doi: 10.1093/biomet/78.3.691

[38] Russell S, Renshaw I, Davids K. Sport arbitration as an emergent process in a complex system: Decision-making variability is a marker of expertise in national-level football referees. J Appl Sport Psychol. 2020;34(3):539–63. doi: 10.1080/10413200.2020.1831651

[39] Voigt L, Friedrich J, Grove P, Heinrich N, Ittlinger S, Iskra M, et al. Advancing judgment and decision-making research in sport psychology by using the body as an informant in embodied choices. Asian J Sport Exerc Psychol. 2023;3(1):47–56. doi: 10.1016/j.ajsep.2022.09.006

[40] Ab Rasid AM, Muazu Musa R, Abdul Majeed APP, Musawi Maliki ABH, Abdullah MR, Mohd Razmaan MA, et al. Physical fitness and motor ability parameters as predictors for skateboarding performance: A logistic regression modelling analysis. PLoS One. 2024;19(2):e0296467. doi: 10.1371/journal.pone.0296467 38329954 PMC10852284

[41] Musa RM, Majeed APPA, Ab Rasid AM, Abdullah MR. Data mining and machine learning in sports: Success metrics for elite goalkeepers in European football leagues. Singapore: Springer; 2024.

[42] Rajšp A, Fister Jr I. A systematic literature review of intelligent data analysis methods for smart sport training. Appl Sci. 2020;10:3013.

[43] Jewell RT. Estimating Demand for aggressive play: the case of English Premier League Football. Int J Sport Financ. 2009;4(3):192–210. doi: 10.1177/155862350900400304

[44] Barnes C, Archer DT, Hogg B, Bush M, Bradley PS. The evolution of physical and technical performance parameters in the English Premier League. Int J Sports Med. 2014;35(13):1095–100. doi: 10.1055/s-0034-1375695 25009969

[45] Yi Q, Liu H, Nassis GP, Gómez M-Á. Evolutionary Trends of Players’ Technical Characteristics in the UEFA Champions League. Front Psychol. 2020;11:1032. doi: 10.3389/fpsyg.2020.01032 32612550 PMC7308470

[46] Abdullah MR, Musa RM, Kosni NA, Maliki A, Haque M. Profiling and distinction of specific skills related performance and fitness level between senior and junior Malaysian youth soccer players. Int J Pharm Res. 2016;8:64–71.

[47] Abdullah MR, Maliki A, Musa RM, Kosni NA, Juahir H, Mohamed SB. Identification and comparative analysis of essential performance indicators in two levels of soccer expertise. Int J Adv Sci Eng Inf Technol. 2017;7:305–14.

[48] Muazu Musa R, P P Abdul Majeed A, Abdullah MR, Ab Nasir AF, Arif Hassan MH, Mohd Razman MA. Technical and tactical performance indicators discriminating winning and losing team in elite Asian beach soccer tournament. PLoS One. 2019;14(6):e0219138. doi: 10.1371/journal.pone.0219138 31247012 PMC6597106

[49] O’Brien KA, Mangan J. The issue of unconscious bias in referee decisions in the National Rugby League. Front Sport Act Living. 2021;260. 10.3389/fspor.2021.739570 PMC8514768 34661100

[50] Nazarudin MN, Abdul Majeed APP, Husin Musawi Maliki AB, Abdullah MR, Kuan G, Muazu Musa R. Disciplinary measures defining referee activity in top-European football leagues: A cross-sectional investigation. Heliyon. 2024;10(3):e25402. doi: 10.1016/j.heliyon.2024.e25402 38352766 PMC10861984

[51] Hafeez A, Hafeez U, Amin A, Hasan S. VAR technology in English football: Implications of intervening in a fast-moving game. Int Sport Stud. 2022;44.

[52] Mascarenhas DRD, Collins D, Mortimer P. The Art of Reason versus the Exactness of Science in Elite Refereeing: Comments on Plessner and Betsch (2001). J Sport Exerc Psychol. 2002;24(3):328–33. doi: 10.1123/jsep.24.3.328 28682206

[53] MacMahon C, Parrington L, Pickering T, Aitken B, Schücker L. Understanding the effects of cognitive tasks on physical performance: a constraints framework to guide further research. Int Rev Sport Exerc Psychol. 2021;16(1):584–618. doi: 10.1080/1750984x.2021.1907854

[54] Işın A, Yi Q. Does video assistant referee technology change the magnitude and direction of home advantages and referee bias? A proof-of-concept study. BMC Sports Sci Med Rehabil. 2024;16(1):21. doi: 10.1186/s13102-024-00813-9 38238850 PMC10797986

[55] Abbate C, Cross J, Uhrig R. Video assistant referee and home field advantage: implications for referee bias. South Econ J. 2025;91:1176–96.

[56] Sánchez R, García-de-Alcaraz A. Crowd effects, territoriality and home advantage: a sociological explanation. In: García MS, editor. Home Advantage in Sport. London: Routledge; 2021. pp. 51–63.

